# Hiding in Plain Sight: Mining Bacterial Species Records for Phenotypic Trait Information

**DOI:** 10.1128/mSphere.00237-17

**Published:** 2017-08-02

**Authors:** Albert Barberán, Hildamarie Caceres Velazquez, Stuart Jones, Noah Fierer

**Affiliations:** a Department of Soil, Water, and Environmental Science, University of Arizona, Tucson, Arizona, USA; b Department of Biological Sciences, University of Notre Dame, Notre Dame, Indiana, USA; c Department of Ecology and Evolutionary Biology, University of Colorado, Boulder, Colorado, USA; d Cooperative Institute for Research in Environmental Sciences, University of Colorado, Boulder, Colorado, USA; University of British Columbia

**Keywords:** pH, phenotypes, phylogeny, salinity, traits

## Abstract

Cultivation in the laboratory is essential for understanding the phenotypic characteristics and environmental preferences of bacteria. However, basic phenotypic information is not readily accessible. Here, we compiled phenotypic and environmental tolerance information for >5,000 bacterial strains described in the *International Journal of Systematic and Evolutionary Microbiology* (IJSEM) with all information made publicly available in an updatable database. Although the data span 23 different bacterial phyla, most entries described aerobic, mesophilic, neutrophilic strains from *Proteobacteria* (mainly *Alpha*- and *Gammaproteobacteria*), *Actinobacteria*, *Firmicutes*, and *Bacteroidetes* isolated from soils, marine habitats, and plants. Most of the routinely measured traits tended to show a significant phylogenetic signal, although this signal was weak for environmental preferences. We demonstrated how this database could be used to link genomic attributes to differences in pH and salinity optima. We found that adaptations to high salinity or high-pH conditions are related to cell surface transporter genes, along with previously uncharacterized genes that might play a role in regulating environmental tolerances. Together, this work highlights the utility of this database for associating bacterial taxonomy, phylogeny, or specific genes to measured phenotypic traits and emphasizes the need for more comprehensive and consistent measurements of traits across a broader diversity of bacteria.

**IMPORTANCE** Cultivation in the laboratory is key for understanding the phenotypic characteristics, growth requirements, metabolism, and environmental preferences of bacteria. However, oftentimes, phenotypic information is not easily accessible. Here, we compiled phenotypic and environmental tolerance information for >5,000 bacterial strains described in the *International Journal of Systematic and Evolutionary Microbiology* (IJSEM). We demonstrate how this database can be used to link bacterial taxonomy, phylogeny, or specific genes to measured phenotypic traits and environmental preferences. The phenotypic database can be freely accessed (https://doi.org/10.6084/m9.figshare.4272392), and we have included instructions for researchers interested in adding new entries or curating existing ones.

## Abstract (precis)

Cultivation in the laboratory is key for understanding the phenotypic characteristics, growth requirements, metabolism, and environmental preferences of bacteria. However, oftentimes, phenotypic information is not easily accessible. Here, we compiled phenotypic and environmental tolerance information for >5,000 bacterial strains described in the *International Journal of Systematic and Evolutionary Microbiology* (IJSEM). We demonstrate how this database can be used to link bacterial taxonomy, phylogeny, or specific genes to measured phenotypic traits and environmental preferences. The phenotypic database can be freely accessed (https://doi.org/10.6084/m9.figshare.4272392), and we have included instructions for researchers interested in adding new entries or curating existing ones.

## INTRODUCTION

Cultivation in the laboratory is one of the most valuable strategies available for describing the morphological characteristics, growth requirements, metabolic capabilities, and environmental preferences of bacterial strains (1). However, cultivation is often overlooked in the era of high-throughput molecular methods, where increasingly more focus is placed on sequencing genomes or metagenomes instead of describing the phenotypic characteristics of axenic cultures (2). This recent increase in the number of bacteria with sequenced genomes has far outpaced the rate at which new bacterial strains are being cultivated and formally described. Therefore, only 30% of bacterial and archaeal type strains have an associated public genome project (3). At the same time, we often lack phenotypic and environmental tolerance data for many of the bacterial genomes being deposited in sequence databases (4). Either the phenotypic data were never collected or reported, or this information has not been compiled into searchable databases to permit downstream analyses and integration with genomic information.

Although genomic analyses of uncultivated microorganisms are undoubtedly valuable (5), they are no panacea, as it can often be difficult to predict the realized phenotypes of bacteria from the presence or absence of particular genes or inferred metabolic pathways from genomic data alone (6, 7). For example, 27% of the differences observed in the growth yield of *Escherichia coli* strains could not be explained by the presence/absence of degradation pathways (8). As another example, because the ammonia monooxygenase gene (*amoA*) is homologous to the methane monooxygenase gene (*pmoA*), the presence of an *amoA* gene or *pmoA*-like genes could indicate that a bacterium is capable of either methane oxidation, ammonia oxidation, or both—two completely different biogeochemical processes (9). These limitations are compounded by the fact that a large fraction of bacterial genes are of undetermined function, and many genes that are annotated have no experimentally validated function and thus may be annotated incorrectly (10).

We acknowledge that cultivation-based studies of bacterial strains have their own set of limitations (11). Many bacteria are difficult to culture (12); observed phenotypes of a bacterial strain growing under laboratory conditions could be very different from the phenotypes of the strain in its natural habitat (13). Additionally, laboratory assays often do not capture the phenotypic information that is likely most relevant to understanding the ecological and physiological attributes of bacterial strains (14). Nevertheless, compiling phenotypic information from cultivated bacterial strains and integrating this information with genomic or marker gene data are critical for advancing the field of microbial ecology. In particular, a database of phenotypic information would (i) improve our ability to assess the phylogenetic breadth and coherence of bacterial traits (15, 16); (ii) help to identify genes, gene categories, and metabolic pathways associated with specific phenotypic traits or growth requirements (17–19); (iii) improve assessments of functional tradeoffs in microbial communities (20); (iv) link observed changes in the abundances of taxa determined via 16S rRNA gene sequencing to phenotypic attributes (21); and (v) divide bacterial taxa into ecologically relevant functional groups (22, 23).

One of the best sources of phenotypic information on cultivated bacteria is the *International Journal of Systematic and Evolutionary Microbiology* (IJSEM). With over 39,000 articles published since 1951, this journal has been the official journal of record for naming bacteria and describing strain characteristics (24). In short, there is clearly a wealth of relevant information on bacterial strains contained within the pages of IJSEM, but this information is not currently readily searchable, and to our knowledge, there have been no comprehensive attempts to collate information from the journal entries in a manner that would allow for downstream analyses and broader use of this information by microbiologists and microbial ecologists (but see BacDive [25] for a manually curated web portal with information on cultured bacterial and archaeal strains and also FAPROTAX [26] for a tool to map prokaryotic clades to ecologically relevant functions).

Here, we outline an ongoing effort to compile and curate selected phenotypic information from bacterial strains described in IJSEM. To date, we have gathered data from a total of >5,000 bacterial strains spanning 23 different phyla with associated information on key phenotypic characteristics for most of these strains. We demonstrate how this database can be used to explore the diversity of bacterial phenotypes, determine the phylogenetic coherence of phenotypic traits, and link gene content to environmental preferences.

## RESULTS AND DISCUSSION

### Description of the phenotypic database.

**FIG 1:**
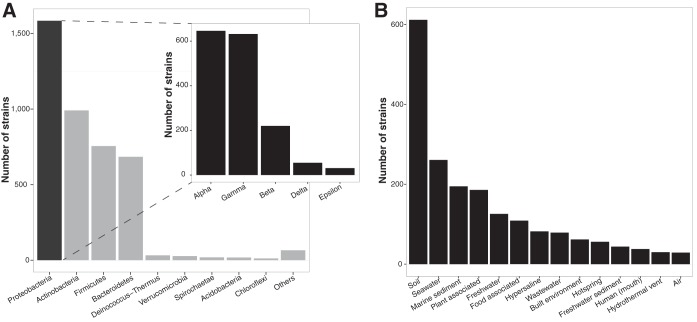
Taxonomic distribution (A) and habitat distribution (B) of the >4,000 bacterial strains present in the phenotype database. The inset in panel A shows the strain representation of the major proteobacterial subgroups in the database. Note that in panel B the habitat is the environment from which each strain was originally isolated (if reported) and may not accurately reflect where those strains may be most abundant.

We collected phenotypic information for 5,130 bacterial strains described in papers published in the *International Journal of Systematic and Evolutionary Microbiology* (IJSEM) from 2004 to 2014 (Table 1). The information compiled was not distributed evenly across the different categories. For example, IJSEM entries described mostly strains from four bacterial phyla: *Proteobacteria* (mainly *Alpha*- and *Gammaproteobacteria*), the Gram-positive *Actinobacteria* and *Firmicutes*, and *Bacteroidetes* (Fig. 1A). While these four phyla account for ~90% of all cultivated bacteria (27), other phyla commonly observed using cultivation-independent techniques like *Acidobacteria*, *Chloroflexi*, *Gemmatimonadetes*, or *Verrucomicrobia* tend to be systematically underrepresented in culture collections (12, 28). Similarly, most bacterial strains with a valid habitat entry were recovered from three main environments: soil, marine habitats, and plants (Fig. 1B). However, we should interpret these results with caution, as often the habitat of isolation might not correspond to the habitats where those strains might be found, even abundant. For example, *Escherichia coli* and other human commensals can be frequently recovered from polluted waters (29), while soil bacteria like *Pseudomonas aeruginosa* can occasionally become opportunistic pathogens and thus can be isolated from animal and plant tissues (30).

**TABLE 1:** Information compiled from the *International Journal of Systematic and Evolutionary Microbiology* (IJSEM) publications

Category	Components
Ancillary data	Yr of publication, article digital object identifier (doi), taxonomic nomenclature, culture collection code
Morphology/phenotype	Gram stain status, cell length, cell width, cell shape, cell aggregation, motility, spore and pigment formation
Metabolism	General metabolism, sole carbon substrate use, BIOLOG information available
Environmental preferences	Habitat of isolation; oxygen requirement; range and optimum for pH, temp, and salt
Sequence data	GC content, 16S rRNA accession no., genome accession no.

**FIG 2:**
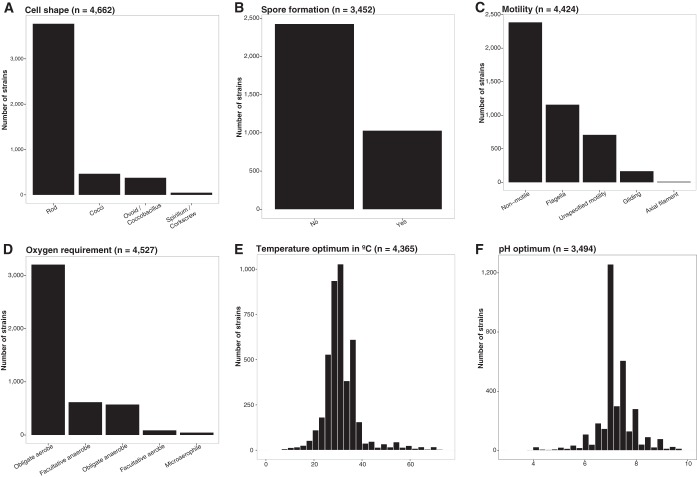
Distribution of selected traits across the >4,000 strains in the most recent version of the database, including cell shape (A), spore formation (B), motility (C), oxygen requirements (D), temperature optimum (E), and pH optimum (F). The number of strains with information for a particular trait is indicated in parentheses.

We also found that most of the IJSEM entries were from aerobic, mesophilic, neutrophilic bacteria (Fig. 2). This likely reflects the cultivation approaches that are most widely used, and these results do not necessarily imply that most environmental bacteria grow best under those conditions. The range in commonly used culture conditions reflects logistical and historical constraints in cultivation-based studies, more so than any attempt to reproduce the range of environmental conditions that bacteria experience *in situ* (31). Besides this issue, bacterial strain descriptions rarely include information on the range of possible environmental conditions under which a given bacterial strain can grow. For example, it is often reported that a strain grows at pH 7, but it remains unclear if that is its optimal pH for growth and how its growth at pH 7 might compare to growth at pH 4. The same problem is apparent with temperature, as strains are often reported to grow at 30°C (Fig. 2E and F), a common temperature in most laboratory incubators, but it is unclear if they would grow better or worse at other temperatures. Additionally, although detailed guidelines for the characterization of bacteria exist (24), not all phenotypic traits and environmental preferences are measured in a completely consistent manner. Thus, caution must be used when using information collected from bacterial isolates growing under laboratory conditions to infer the ecological attributes of these same bacteria in their natural habitat.

Many bacteria are not readily cultivable in the laboratory. This so-called “great plate count anomaly” arose from the observation that microscopic cell counts were significantly larger than the number of colonies growing on solid medium (32). One hypothesis as to why most environmental microbes are not cultivable is that the appropriate growth conditions are unknown and complex or not feasible to replicate in the laboratory. Likewise, many taxa may simply be difficult to cultivate under laboratory conditions because they replicate slowly (33). New cultivation techniques, including the use of very dilute medium to select for oligotrophs, coculturing with other bacteria, and novel microcultivation technologies, have and will continue to increase the taxonomic breadth of cultivated bacteria (31). For example, a recent study showed that the common practice of autoclaving agar and phosphate buffer together to prepare solid growth medium inhibits the cultivation of environmental bacteria (11). These biases have been long known (32), and it is acknowledged that traditional cultivation techniques will tend to favor faster-growing, cosmopolitan distributed microorganisms with potentially broad metabolic capabilities (27).

### Phylogenetic signal of phenotypic traits.

**FIG 3:**
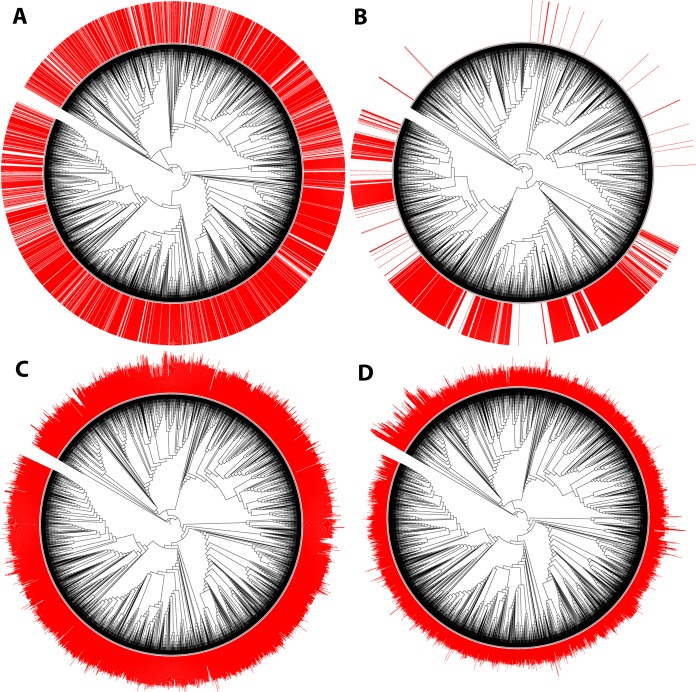
Phylogenetic signal of selected traits: presence of pigment (A), spore formation (B), pH optima (C), and temperature optima (D). For categorical variables (A and B), the red columns indicate presence. For continuous variables (C and D), the red columns indicate the reported value.

Besides a general description of the database and its biases and limitations, we demonstrate how this information could be useful for evolutionary microbiologists and microbial ecologists. First, we had near-full-length 16S rRNA gene sequences for 4,188 bacterial strains, and we used this marker gene information to assess the evolutionary relationships between strains and calculate the phylogenetic signal (i.e., similarity among species related to phylogenetic relatedness) of categorical and continuous traits (Table 2). While widespread traits like pigment formation had weak phylogenetic signal (Fig. 3A), morphological traits like Gram stain result, spore formation (Fig. 3B), or cell shape tended to show the strongest phylogenetic signal. Salinity and pH optima did not exhibit a significant phylogenetic signal across bacterial strains (Fig. 3C). Previous studies have observed a phylogenetic signal in salinity tolerance across aquatic bacterial taxa (34); such a signal may be more apparent when comparing salinity tolerances across specific lineages from a subset of environments or in studies that capture uncultivated as well as cultivated taxa. Temperature optimum showed a weak phylogenetic signal (Fig. 3D), mainly driven by the adaptation to extremely hot environments of deep-branching phyla, including the *Aquificales* and *Thermotogae* (35).

**TABLE 2:** Phylogenetic signal of bacterial traits

Trait	Type^a^	Phylogenetic signal^b^
Spore	Categorical	**1.225**
Pigment	Categorical	**0.219**
Shape (rod)	Categorical	**0.628**
Shape (coccus)	Categorical	**0.703**
Aggregation (chain)	Categorical	**0.182**
Gram stain	Categorical	**1.516**
Flagella	Categorical	**0.495**
Aerobe	Categorical	**0.575**
Anaerobe	Categorical	**0.593**
Temp preference	Continuous	**0.226**
pH preference	Continuous	0.006
Salinity preference	Continuous	0.023

a −*D* + 1 for categorical, Blomberg’s *K* for continuous.

b Values in bold are significant (*P* < 0.05).

Overall, our results confirm three previous general observations. First, most bacterial traits tend to show a significant phylogenetic signal, but the signal is often weak and the ability to predict a phenotypic trait from phylogeny alone will vary greatly depending on the trait of interest (7). Second, complex traits like spore formation or photosynthesis are more likely to be highly conserved (15, 16), with these phenotypes often predictable at even coarse levels of taxonomic resolution. Third, the phylogenetic signal tends to be weak for environmental preferences (16), including pH, temperature, and salinity optima. Thus, predicting the environmental preferences from phylogenetic information alone remains difficult, particularly for lineages that are not well described. Together, this work adds to the large body of evidence that, due to the promiscuity of horizontal gene transfer, convergent evolution, and gene loss, bacterial taxa with highly similar 16S rRNA sequences can potentially display very distinct phenotypic characteristics (36). Any attempt to predict phenotype from phylogeny or taxonomy alone (including the widely used PICRUSt approach [37]) should be pursued with caution.

### Linking genomic information to pH and salinity optima.

We were able to find whole-genome data for 29% of the database strain entries to link gene content and the presence/absence of gene categories and metabolic pathways to pH optima (67% of strains with a genome reported a value) and salinity optima (52% of strains with a genome reported a value) using an enrichment analysis based on logistic regression. Recent work has linked gene expression profiles and genomic attributes to bacterial phenotypes (38, 39), trophic strategies in marine bacteria (18), microbial growth rates (17), bacterial life history strategies (19, 40), and even habitat breadth in soil bacteria (21). We wanted to determine if we could also use genomic information to predict pH and salinity preferences, traits that are important given that pH and salinity are key factors that often shape bacterial communities in a wide range of environments, including soil (41), aquatic environments (42), and human skin (43). Likewise, given that there are many uncultivated (or difficult-to-culture) taxa for which we can now readily obtain genomes via single-cell or metagenomic sequencing (2, 5), estimating the pH and salinity preferences from genomes of uncultivated taxa will aid in the design of medium conditions for more effective cultivation.

Previous research shows that adaptation or acclimatization to saline or extreme pH environments is often related to the complement of cell surface transporters that a bacterium possesses or expresses (44–46). Our KEGG ortholog (KO) enrichment analysis strongly supports this conventional wisdom. Of the 33 and 14 enriched KOs for pH and salinity, respectively, 26 (79%) and 9 (64%) were known to mediate a transport function in bacteria. Also, the sign of the logistic regression coefficients was consistent with selection for growth under high salinity or low pH (Table 3). We observed a tendency for the absence of a high-affinity potassium transport system (*kdpABC*; K01546 to K01548) to correlate with a higher salinity optimum (47). We also saw a tendency for strains with higher pH optima to encode an Na^+^/H^+^ antiporter (*mnhACDEFG*), previously suggested to be adaptive under alkaline conditions (46, 48). Interestingly, we observed several KOs that were correlated strongly with pH but encoded functions typically associated with salinity tolerance. For example, we found that KOs encoding synthesis of the osmoprotectant ectoine (K06718 and K06720) were correlated with pH but not salinity optima (Table 3). Recent work suggests that ectoine may have a role in stabilizing enzymes at extreme pH values (49). Our result indicates that pH homeostasis may be another role for ectoine in bacteria. Similarly, we observed significant correlations between two KOs related to compatible solute transport (K02168 and K03451) and pH (Table 3), suggesting that the acquisition of compatible solutes may also have a secondary role in pH tolerance.

**TABLE 3:** Putative genomic markers associated with pH and salinity optima

KO ID^a^	Optimum	Description^c^	Sign of coefficient	TCDB^b^ present
K01546	Both	K^+^-transporting ATPase ATPase A chain	−	Yes
K01547	Both	K^+^-transporting ATPase ATPase B chain	−	Yes
K01548	Both	K^+^-transporting ATPase ATPase C chain	−	Yes
K03310	Both	Alanine or glycine:cation symporter, AGCS family	+	Yes
K03499	Both	Trk system potassium uptake protein	+	Yes
K07301	Both	Cation:H^+^ antiporter	+	Yes
K08974	Both	Putative membrane protein	+	No
K03543	pH	Membrane fusion protein, multidrug efflux system	−	Yes
K03446	pH	MFS transporter, DHA2 family, multidrug resistance protein	−	Yes
K08677	pH	Kumamolisin	−	No
K07799	pH	Membrane fusion protein, multidrug efflux system	−	Yes
K06045	pH	Squalene-hopene/tetraprenyl-beta-curcumene cyclase	−	Yes
K15495	pH	Molybdate/tungstate transport system substrate-binding protein	−	Yes
K15496	pH	Molybdate/tungstate transport system permease protein	−	Yes
K14393	pH	Cation/acetate symporter	+	Yes
K02168	pH	Choline/glycine/proline betaine transport protein	+	Yes
K07393	pH	Putative glutathione *S*-transferase	+	No
K06718	pH	l-2,4-Diaminobutyric acid acetyltransferase	+	No
K06720	pH	l-Ectoine synthase	+	No
K09908	pH	Uncharacterized protein	+	No
K06213	pH	Magnesium transporter	+	Yes
K05565	pH	Multicomponent Na^+^:H^+^ antiporter subunit A	+	Yes
K05567	pH	Multicomponent Na^+^:H^+^ antiporter subunit C	+	Yes
K05568	pH	Multicomponent Na^+^:H^+^ antiporter subunit D	+	Yes
K05569	pH	Multicomponent Na^+^:H^+^ antiporter subunit E	+	Yes
K05570	pH	Multicomponent Na^+^:H^+^ antiporter subunit F	+	Yes
K05571	pH	Multicomponent Na^+^:H^+^ antiporter subunit G	+	Yes
K14683	pH	Solute carrier family 34 (sodium-dependent phosphate cotransporter)	+	Yes
K14445	pH	Solute carrier family 13 (sodium-dependent dicarboxylate transporter), member 2/3/5	+	Yes
K03451	pH	Betaine/carnitine transporter, BCCT family	+	Yes
K03308	pH	Neurotransmitter:Na^+^ symporter, NSS family	+	Yes
K08714	pH	Voltage-gated sodium channel	+	Yes
K03826	pH	Putative acetyltransferase	+	No
K03975	Salinity	Membrane-associated protein	−	Yes
K08223	Salinity	MFS transporter, fosmidomycin resistance protein	−	Yes
K07646	Salinity	Two-component system, OmpR family, sensor histidine kinase KdpD	−	No
K03549	Salinity	KUP system potassium uptake protein	−	Yes
K03699	Salinity	Putative hemolysin	−	No
K02276	Salinity	Cytochrome *c* oxidase subunit III	+	No
K07160	Salinity	UPF0271 protein	+	No

a KO ID, entry in KEGG ortholog (KO) database.

b TCDB indicates whether the enriched KO was included in the Transporter Classification Database.

c Abbreviations: AGCS, alanine or glycine cation symporter; MFS, major facilitator superfamily; BCCT, betaine carnitine choline transporter; NSS, neurotransmitter sodium symporter; KUP, K uptake permease.

Although we overwhelmingly enriched for transport proteins, the nontransporter KOs also revealed an imprint of osmotic or pH-based selection. For example, one of the nontransporter enriched KOs for salinity optimum (K07646) is a well-characterized, sensor histidine kinase (*kdpD*) that regulates expression of a high-affinity potassium transport operon (*kdpABC*) (47). All of these genes (*kdpD* and *kdpABC*) were negatively associated with salinity optimum across the strains in our database (Table 3). Further, a nontransporter KO enriched in our pH optimum models (K08677; negatively associated with pH optimum) encodes kumamolisin, which is a peptidase known to have high activity under low-pH conditions (50, 51).

Together, these analyses serve as simple examples of the opportunity to link ecological traits to genome content through the use of a bacterial phenotypic trait database. We observed a number of putative genotype-phenotype links that are consistent with previous species-specific genetic studies, but we also identified a number of previously uncharacterized proteins that should be further explored as playing a role in phenotypic adaptation. Although we were able to infer pH and salinity preferences of cultured bacterial strains based on a few functional categories, further experimental work is required to determine how well these pH and salinity markers can predict pH and salinity preferences in the environment.

### Future research.

Trait-based approaches have advanced our mechanistic understanding of ecological processes from populations to ecosystems (52). Along these lines, the Unified Microbiome Initiative recently stated: “Simply knowing which genes are present in a microbial population, without understanding their physical linkage, precludes organism-based insights into community function and dynamics” (53). That being so, cultivation of bacteria is essential for understanding bacterial phenotypes and their ecological attributes. However, phenotypic information is not readily accessible and phenotype is often difficult to infer from taxonomic, phylogenetic, or genomic information alone. Here, we described the phenotypic and environmental tolerance information from >5,000 bacterial strains described in the *International Journal of Systematic and Evolutionary Microbiology* (IJSEM). We encourage other researchers to curate the initial version of the phenotypic database (https://doi.org/10.6084/m9.figshare.4272392) and also to contribute with new entries.

We demonstrated how this phenotypic database from IJSEM publications can be used to explore the diversity of bacterial traits, assess the phylogenetic signal of phenotypic traits and environmental preferences, and link genomic attributes to pH and salinity optima. We believe that the database described here will ultimately be of value to researchers exploring bacterial functional trait tradeoffs, assessing community-aggregated traits derived from metagenomics and their relationship with ecosystem functions (20), informing environmental surveys in search of novel strains to isolate, and dividing bacterial taxa into ecological guilds based on phenotypic characteristics (22, 23).

## MATERIALS AND METHODS

### Database compilation and curation.

The *International Journal of Systematic and Evolutionary Microbiology* (IJSEM) is the official publication of the International Committee on Systematics of Prokaryotes and the Bacteriology and Applied Microbiology Division of the International Union of Microbiological Societies and the official journal of record for novel bacterial and archaeal taxa (http://ijs.microbiologyresearch.org/content/journal/ijsem/). We manually searched IJSEM articles to extract phenotypic, metabolic, and environmental tolerance data of bacterial strains described in the notification list from 2004 to 2014 (Table 1). Although not all information could be retrieved for each bacterial strain, this subset of characteristics provided relevant information on the morphological, metabolic, and ecological attributes of the described strains and tended to be reported in a consistent manner for most strains. We note that we did not collect all available information reported for each strain. We ignored those phenotypic characteristics that were (i) collected for only a small subset of strains (e.g., cell stoichiometry), (ii) difficult to compare across strains (e.g., reported growth rates on individual medium types), or (iii) deemed to be of limited utility (e.g., specific information on phospholipid-derived fatty acid profiles).

In this initial census, we focused on the most recent entries as they presumably used standardized and state-of-the-art methods and up-to-date taxonomic nomenclature, most strains had easily retrievable 16S rRNA gene sequence data, and many strains also had publicly available genome sequence data available (24). Data were manually collected using Google Forms as variable structure of the articles and inconsistent reporting of relevant information (i.e., phenotypic information tends to be semantically opaque and needs to be interpreted in a biological context) precluded the use of automatic text parsing algorithms (although we acknowledge that human indexing is error prone). For example, articles reporting “nitrate reductase activity found,” “denitrification activity,” “nitrate reductase present,” “positive reduction of nitrate,” “positive nitrate reduction,” “positive for nitrate reductase,” “capable of nitrate reduction,” or “nitrate reducer” all point to the same process of anaerobic growth in the presence of nitrate. That is, authors of taxonomic publications may describe the same or very similar features using different terms across articles or even within the same article. Additionally, some terms are unique for specific taxonomic groups. For example, aggregation in chains is reported both for filamentous cyanobacteria and for growth-rate-dependent chains in stationary-phase cultures of many heterotrophs. However, natural processing algorithms to extract phenotypic data from prokaryotic taxonomic descriptions are an active area of research (54). The generated raw file was curated using automated scripts and manual checks to detect data entry errors, duplicated entries, and format inconsistencies. Raw data and curated data can be freely accessed in figshare (https://doi.org/10.6084/m9.figshare.4272392), and we have included specific instructions for outside users interested in adding to this database.

### Phylogenetic signal analyses.

From the total of 5,130 bacterial strains, we associated valid, complete, and nonduplicated 16S rRNA gene entries with ~4,200 strains. To infer the evolutionary relationships among the bacterial strains, we first aligned the complete 16S rRNA gene sequences using PyNAST (55) with the Greengenes database (56) as a template. The resulting multiple sequence alignment was trimmed to remove positions which are gaps in every sequence, and a phylogenetic tree was reconstructed with the FastTree approximate maximum-likelihood algorithm (57) using the midpoint method for rooting.

We measured the phylogenetic signal of continuous traits with Blomberg’s K (58) using the function phylosignal in the Picante R package (59). This metric expresses the deviation from a Brownian motion evolutionary model (*K* = 0 corresponds to no phylogenetic signal; *K* > 0 corresponds to a trait that is more conserved than expected by chance). For categorical traits, we used the *D* value using the function phylo.D (60) in the caper R package. This metric compares observed sister-clade differences against those expected for a random phylogeny. In order to compare with Blomberg’s *K*, we transformed the *D* value into −*D* + 1 (−*D* + 1 = 0 corresponds to no phylogenetic signal; −*D* + 1 > 0 corresponds to a conserved trait) (16). Statistical significance was estimated by permuting phenotypic trait values across the tips of the phylogenetic tree 1,000 times.

### Association between genomic attributes and environmental preferences.

We matched the associated complete 16S rRNA gene sequences against a 16S rRNA database from sequenced bacterial genomes at >99% identity and >95% coverage. For the 29.4% of strains that had publicly available closely related genome sequence data, we downloaded genomic data and annotated functional gene information from the Integrated Microbial Genomes (IMG) database (https://img.jgi.doe.gov/) (61). We used the 754 strains with available closely related genomes to provide a simple demonstration of the utility of linking phenotypic traits from our database to genomic information. We selected pH and salinity optima for this purpose because these were continuous traits that displayed no phylogenetic signal (Table 2). When pH and salinity were exclusively reported as a range, we calculated the optimum as the equidistant value between the reported maximum and minimum. Of the 754 bacterial strains in our database that had a genome sequence, 503 had a known pH optimum value and 391 had a known salinity optimum. To identify putative genomic markers of these traits, we conducted a simple enrichment analysis using logistic regression. We used KEGG ortholog (KO) presence-absence in each of the strain genomes (http://www.genome.jp/kegg/), accessed from IMG, as our response variable. The probability of the presence of each KO in a strain’s genome was modeled as a function of the strain’s salinity or pH optimum. The presence of a significant salinity or pH coefficient in the logistic regression, after Bonferroni correction, indicated a putative link between a KO and the phenotypic trait. We selected an overall alpha value of 0.05, meaning that after Bonferroni correction for the 6,889 model fits (one for each KO in the IMG data set), the significance cutoff for any individual logistic regression was 7.3e−6. Because previous work has shown the involvement of cell surface transporters in adaptation and acclimatization of individual bacteria strains to both salinity and pH (44, 45), we classified enriched KOs as transporters based upon their inclusion in the Transporter Classification Database (http://tcdb.org/) (62).
